# Dietary galactose exacerbates autoimmune neuroinflammation *via* advanced glycation end product-mediated neurodegeneration

**DOI:** 10.3389/fimmu.2024.1367819

**Published:** 2024-08-09

**Authors:** Stefanie Haase, Kristina Kuhbandner, Florian Mühleck, Barbara Gisevius, David Freudenstein, Sarah Hirschberg, De-Hyung Lee, Stefanie Kuerten, Ralf Gold, Aiden Haghikia, Ralf A. Linker

**Affiliations:** ^1^ Department of Neurology, University Hospital Regensburg, Regensburg, Germany; ^2^ Department of Neurology, University Hospital Erlangen, Friedrich-Alexander-University Erlangen-Nuremberg, Erlangen, Germany; ^3^ Department of Neurology, St. Josef-Hospital Bochum, Ruhr-University Bochum, Bochum, Germany; ^4^ Institute of Neuroanatomy, Faculty of Medicine, University of Bonn and University Hospital Bonn, Bonn, Germany; ^5^ Department of Neurology, University Medicine Magdeburg, Magdeburg, Germany

**Keywords:** galactose, advanced glycation end products, neuroinflammation, MOG-EAE, oligodendrocytes, human induced primary neurons, multiple sclerosis

## Abstract

**Background:**

Recent studies provide increasing evidence for a relevant role of lifestyle factors including diet in the pathogenesis of neuroinflammatory diseases such as multiple sclerosis (MS). While the intake of saturated fatty acids and elevated salt worsen the disease outcome in the experimental model of MS by enhanced inflammatory but diminished regulatory immunological processes, sugars as additional prominent components in our daily diet have only scarcely been investigated so far. Apart from glucose and fructose, galactose is a common sugar in the so-called Western diet.

**Methods:**

We investigated the effect of a galactose-rich diet during neuroinflammation using myelin oligodendrocyte glycoprotein-induced experimental autoimmune encephalomyelitis (MOG-EAE) as a model disease. We investigated peripheral immune reactions and inflammatory infiltration by *ex vivo* flow cytometry analysis and performed histological staining of the spinal cord to analyze effects of galactose in the central nervous system (CNS). We analyzed the formation of advanced glycation end products (AGEs) by fluorescence measurements and investigated galactose as well as galactose-induced AGEs in oligodendroglial cell cultures and induced pluripotent stem cell-derived primary neurons (iPNs).

**Results:**

Young mice fed a galactose-rich diet displayed exacerbated disease symptoms in the acute phase of EAE as well as impaired recovery in the chronic phase. Galactose did not affect peripheral immune reactions or inflammatory infiltration into the CNS, but resulted in increased demyelination, oligodendrocyte loss and enhanced neuro-axonal damage*. Ex vivo* analysis revealed an increased apoptosis of oligodendrocytes isolated from mice adapted on a galactose-rich diet. *In vitro*, treatment of cells with galactose neither impaired the maturation nor survival of oligodendroglial cells or iPNs. However, incubation of proteins with galactose *in vitro* led to the formation AGEs, that were increased in the spinal cord of EAE-diseased mice fed a galactose-rich diet. In oligodendroglial and neuronal cultures, treatment with galactose-induced AGEs promoted enhanced cell death compared to control treatment.

**Conclusion:**

These results imply that galactose-induced oligodendrocyte and myelin damage during neuroinflammation may be mediated by AGEs, thereby identifying galactose and its reactive products as potential dietary risk factors for neuroinflammatory diseases such as MS.

## Background

Multiple sclerosis (MS) is an autoimmune disease of the central nervous system (CNS) characterized by neuroinflammation and neurodegeneration. Although some progress has been made in understanding its pathogenesis, the mechanisms underlying its neurodegenerative aspects remain largely unclear. In the recent years, it has been shown that neuroinflammatory processes may be influenced by environmental, dietary, and life-style factors, contributing to incidence and disease activity of MS (reviewed in (1, 2)). Since the initial suggestion in the 1950s that diet may have detrimental effects on the course of MS (3), several studies have been published supporting this approach.

We and others could recently demonstrate that a high salt intake promotes neuroinflammation, with *in vivo* experiments showing a worsened course of experimental autoimmune encephalomyelitis (EAE), the murine disease model of MS (4–8). This was associated with enhanced T helper (Th) 17 immunity (4–6, 9, 10) as well as an impaired regulatory T cell (Treg) phenotype and function in high salt conditions (11). Apart from T cells, myeloid cells are also affected by high salt concentrations, leading to an exaggerated activation of pro-inflammatory M1 macrophages while the ability of regulatory M2 macrophages to suppress effector T cell proliferation is reduced (8, 12–15). The clinical relevance of high-salt intake as a risk factor for MS, however, is controversially discussed. While one study revealed an increased disease activity in people with MS due to high salt intake (16), others observed no correlation between salt intake and the risk of MS (17, 18).

An additional dietary factor influencing neuroinflammation are fatty acids. We were able to show that saturated long-chain fatty acids promote the activation of pro-inflammatory Th1 and Th17 cells, resulting in aggravated neuroinflammation during EAE (6, 19). In contrast, the supplementation of the short-chain fatty acid propionic acid (PA) induces an anti-inflammatory milieu in the gut and spleen of EAE diseased mice, increasing the number and functionality of Treg cells (19, 20). This is also clinically relevant as supplementation of PA in therapy-naive MS patients and as an add-on to MS immunotherapy significantly increases functionally competent Treg cells. In line with this observation, MS patients receiving PA show a reduced annual relapse rate together with reduced brain atrophy and a stabilization of disability (21).

Another common element of our so-called Western diet is dairy products, which contain proteins that potentially exert negative effects on neuroinflammatory diseases. Immunization of mice with casein, a protein contained in bovine milk, led to demyelination in the spinal cord, and serologic analyses showed a cross-reactivity of antibodies for casein and myelin-associated glycoprotein, that was also shown to be relevant for patients with MS (22). A similar antibody cross reactivity has been reported for the milk protein butyrophilin and myelin oligodendrocyte glycoprotein (MOG) (23, 24). Besides milk proteins, sugars contained in dairy products might be relevant for pathological processes during neuroinflammation. Here, galactose is of high interest, since it is commonly used in rodent aging models to induce neurodegeneration. To do so, galactose is typically administered intraperitoneally or subcutaneously chronically for up to 16 weeks (25). However, the impact of dietary galactose on neuroinflammatory disease models with a neurodegenerative pathogenesis has not been investigated so far. In fact, there are only few studies investigating the impact of other dietary sugars in the EAE mouse model, such as glucose and sucrose. The intake of these sugars worsened the clinical outcome of EAE, what was attributed to an increased differentiation of Th17 cells (26, 27).

Given the known neurodegenerative effects of galactose treatment in aging models and the potential impact of dietary hexoses on neuroinflammation, we aimed to examine the effect of dietary galactose in the EAE model. Our results demonstrate that a galactose-rich diet exacerbates the disease course in mice by enhancing oligodendrocyte apoptosis and increasing neuro-axonal damage without effects in the immune system.

## Material and methods

### Animal experiments and diet

C57BL/6N mice were bred and housed at the Franz-Penzoldt-Zentrum (FPZ), the animal care facility of the University Erlangen-Nuremberg (Germany), under a 12 h day-night-cycle and standardized environmental conditions receiving normal chow (ssniff V1534-300) and tap water ad libitum. All experiments were in accordance with the German laws for animal protection and were approved by the local ethic committees (AZ 55.2 DMS-2532-2-27). For a sugar-rich diet, mice received normal chow (ssniff V1534-300) and tap water containing 10% of D-galactose ad libitum 14 days prior to EAE induction and throughout the observation period. Control mice received normal tap water throughout the adaption and observation period. Otherwise, the galactose-rich and control diet were completely similar.

### EAE induction

10–12 weeks-old mice were anesthetized and subcutaneously injected with 200 μg myelin oligodendrocyte glycoprotein (MOG_35–55_; Charité, Berlin) and 200 μg Complete Freund’s Adjuvant (Difco) containing 4 mg/ml Mycobacterium tuberculosis (H37RA). Mice received 200 ng Pertussis toxin (List, Germany) intraperitoneally on the day of immunization and two days later. Clinical symptoms were assessed daily according to a 5-point scoring system (4). Body weights of mice receiving either a control or a galactose-rich diet were monitored starting 14 days prior to active immunization and during the course of MOG-EAE.

### Histological analysis

After perfusion with 4% paraformaldehyde (PFA) on days 16 and 34 post immunization (p.i.), spinal cord tissue was embedded in paraffin after 3 h post-fixation in 4% PFA. 4 µm-thick cross sections were deparaffinized in xylene and rehydrated in decreasing concentrations of ethanol before antigen retrieval in 1mM ethylenediaminetetraacetic acid (EDTA) or citrate buffer. Antigens were blocked with 10% BSA/PBS (bovine serum albumin in phosphate-buffered saline) before incubation with the primary antibody overnight at 4°C. Biotinylated secondary antibodies were applied for 60 min at room temperature followed by peroxidase-coupled avidin-biotin complex (ABC Kit, Vector Laboratories, Inc. Burlingame, CA). Reactivity was visualized with diamino-3,3’benzidine (DAB, Vector Laboratories, Inc. Burlingame, CA). Slides were counterstained using Mayer´s hemalaun solution (Merck, Darmstadt, Germany). The following antibodies were used: anti-CD3 1:200 (CD3-12, BIO-RAD Laboratories), anti-Mac-3 1:200 (M3/84, BD Pharmingen), anti-neurite outgrowth inhibitor A (NogoA) 1:200 (polyclonal; Santa Cruz Biotechnology), anti-oligodendrocyte transcription factor 2 (Olig2) 1:500 (rabbit polyclonal, Merck Millipore), anti-2’,3’-cyclic nucleotide 3’ phosphodiesterase (CNPase) 1:1000 (SMI-91; 1:7500, Sternberger Monoclonals via Covance, Princeton, USA) and anti-NeuN 1:100 (MAB2300, Merck Millipore). For analysis of axonal densities, Bielschowsky silver impregnation was used as described recently (20). Spinal cord cross sections were analyzed by a blinded observer using a BX-51 light microscope. Cellular infiltrates were counted in three lesions within each spinal cord segment (cervical, thoracic and lumbar) at 200x magnification within the margin of a stereological grid and quantified per square millimeter of white matter. Analysis of demyelinated areas in the white matter was performed semi-automatically with the help of CellP software. Axonal density was quantified in silver impregnated sections by counting on a 100 µm diameter grid. Data on axons are presented as relative axonal densities per grid (28).

### Electron microscopy

EAE diseased mice were perfused with 4% PFA for 30 min followed by removal of the spinal cord. Segments of the lumbar spinal cord were immediately fixed overnight at 4°C in 4% glutaraldehyde/4% PFA in 0.1 M phosphate buffer/cacodylate buffer, pH 7.2. After washing with phosphate buffer, the tissue was post-fixed in phosphate buffer containing 1% osmium tetroxide and KaFECN(III) followed by washing with distilled water. Specimens were dehydrated in ascending concentrations of ethanol, embedded in epon and polymerized at 65°C for 72 h. Embedded spinal cord samples were cut at 80 nm with an ultra-microtome and analyzed using a Zeiss EM 906 transmission electron microscope (Carl Zeiss, Jena, Germany).

### Isolation of splenocytes

Spleens of mice receiving a galactose-rich diet or control diet were removed on day 10 or day 16 of EAE and disrupted with a 5 ml glass homogenizer. Cells were filtered through a 100 µM cell strainer followed by erythrocyte lysis. Cells were washed with cold Dulbecco’s Phosphate Buffered Saline (DPBS; Gibco *via* Life Technologies, Darmstadt, Germany) and used for flow cytometry analysis or the MOG_35-55_ restimulation assay.

### Isolation of CNS leukocytes

Spinal cord tissue was removed on day 16 of EAE after perfusion with cold DPBS and disrupted with a 5 ml glas homogenizer. Cells were transferred to a Percoll™ (GE Healthcare, Solingen, Germany) density gradient and centrifuged at 800g for 20 min without break. Cells at the interphases were collected, washed with cold DPBS and analyzed by flow cytometry.

### *In vitro* MOG_35-55_ restimulation assay

Splenocytes were seeded at a density of 3x10^6^ cells/cm^2^ in Re-medium. MOG_35-55_ (20 µg/ml) was added for stimulation and the cells were cultured for 48 or 72 hours at 37°C. Supernatants were harvested after 48h and analyzed by enzyme-linked immunosorbent assay (ELISA) for the secretion of IL-17A and IFN-γ (ELISA DuoSet, R&D) according to the manufacturer’s protocol. To assess the proliferation capacity, splenocytes were labelled with e450 proliferation dye (eBioscience) according to the manufacturer’s protocol and cultured in the presence or absence (control) of MOG_35-55._ Proliferation was analyzed 72h later by flow cytometry.

### Flow cytometry

*Ex vivo* obtained CNS leukocytes and splenocytes were analyzed by extra- and intracellular staining. Dead cells were excluded by a fixable viability dye eFluor^®^780 (0.2 μl/test, eBioscience). Nonspecific Fc-mediated interactions were blocked by addition of 0.5 μl anti-CD16/32 (93, eBioscience) for 10 min. For surface staining, cells were stained with the respective fluorochrome- conjugated antibodies for 30 min in PBS. For intracellular cytokine staining, cells were stimulated for 4 h with ionomycin (1 µM; Sigma-Aldrich) and phorbol-12-myristate-13-acetate (PMA; 50 ng/ml; Sigma-Aldrich) in the presence of monensin (2 μM; eBioscience). Cells were stained for surface marker and made permeable by saponin buffer or Fix/Perm (eBioscience) according to the manufacturer’s protocol. Intracellular cytokines were stained with the respective fluorochrome conjugated antibodies for 30-45 min. The following antibodies were used: αCD4-FITC (RM4-5, eBioscience), αCD11b-APC/PE (M1/70, Biolegend), αCD11c-FITC/Pe-Cy7 (HL3, BD Pharmingen), αCD25-APC/BV421 (PC61, BioLegend), αCD44-PE (IM-7, Biolegend), αCD69-BV421 (H1.2F3, Biolegend), αFoxP3-PE/Pe-Cy7 (FJK-16s, eBiosciences), αIL-17A-PE/Pe-Cy7 (eBio17B7, eBioscience), αIFN-γ-APC (XMG1.2, BD Biosciences). Cells were measured with FACSCanto II from BD Biosciences and data were analyzed using FlowJo software (BD Biosciences).

### Preparation of galactose-derived AGE-BSA

Glycation was performed as described previously (29). Shortly, 10 mg/ml BSA, 500 mM galactose, 1 U/ml penicillin/streptomycin and 1 mM EDTA were dissolved in PBS and filtered through a 0.22 µm pore filter before incubated under sterile conditions at 37°C in the dark over a period of 12 weeks. To check for changes over time, aliquots were taken weekly. Until further analysis, solutions were stored at -20°C. Finally, all aliquots were dialyzed against PBS at 4°C to stop the reaction by removal of unbound sugars. AGEs then were stored in sterile tubes at 4°C. For the preparation of control-BSA, this procedure was performed without the addition of galactose.

### Measurement of AGE autofluorescence

Given the special fluorescence characteristics and absorption spectra of some AGE products, auto-fluorescence of pentosidine, a common AGE variant was determined to evaluate the formation of AGEs. Therefore, total protein content was determined by BCA assay (Thermo Scientific) and sample concentrations were adjusted to 1 mg/ml. Arbitrary fluorescent units were measured with a fluorescence plate reader (Victor Multilabel plate reader, Perkin Elmer) at an excitation wavelength of 355 nm and an emission wave length of 460 nm. Fluorescence of PBS alone was subtracted from each data set and results are presented as fluorescent units.

### Oligodendroglial cell apoptosis

O4+ cells were isolated via magnetic cell separation (MACS) from brains of C57BL/6N mice on day 20 of EAE either receiving control or a galactose-rich diet. The cells were stained with the fixable viability dye eFluor^®^780 (0.2 μl/test, eBioscience) for 20 min. Nonspecific Fc-mediated interactions were blocked by addition of 0.5 μl anti-CD16/32 (93, eBioscience) for 10 min before the addition of αO4-APC (130-109-153, Milteniy Biotec) for 30 min. The cells were then labeled with Annexin-V FITC (Invitrogen) according the manufacturers’ instruction. The frequency of Annexin-V^+^ apoptotic cells in O4+ cells was analyzed by flow cytometry.

### Isolation and culture of mixed glial cells

After dissection of cortices from day 1-2 postnatal mouse pups, MGC were isolated using the Neural Tissue Dissociation Kit (Miltenyi Biotech) according to the manufacturer’s protocol. Obtained MGC were re-suspended in 500 µl MGC media containing DMEM (Gibco) supplemented with 1% penicillin/streptomycin (PAN-Biotech GmBH) and 10% heat inactivated horse serum per brain. Cells were seeded in 10 cm-culture dishes pre-coated with poly-D-lysine/laminin (Sigma) and incubated at 37°C and 5% CO_2._ Media was replaced every 2 days. After 14 days in culture, MGC were processed for isolation of oligodendrocytes.

### Isolation and culture of oligodendrocytes

After 14 days in culture, MGC were harvested and labelled with αO4-antibody (Miltenyi Biotech) for separation via magnetic cell sorting (MACS) according to the manufacturer’s instructions. For immunocytochemical analysis, O4^+^ cells were plated on poly-D-lysine -coated cover slips at a density of 5-8 x 10^5^ cells per 4-well chamber and kept at 37°C and 5% CO_2_. Half of the media containing MACS Neuro-Medium (Miltenyi Biotech) supplemented with 2% MACS NeuroBrew-21 (Miltenyi Biotech), 1% L-Glutamine (PAN-Biotech GmBH), 1% penicillin/streptomycin, fibroblast growth factor (FGF, 10 ng/ml, Miltenyi Biotech) and platelet-derived growth factor-aa (PDGF-AA, 10 ng/ml, PeproTech) was changed every other day. Cells were either treated with 1.5 mM galactose for 72 h, or with 50 µM AGE-BSA or control-BSA for 72 h.

### Immunocytochemical analysis

Oligodendrocytes were washed in PBS and fixed in 1% PFA for 30 min at room temperature. After blocking with 10% BSA+0.1% Triton-X, cells were incubated with primary antibodies over night at 4°C. Cells were incubated with secondary antibodies for 1 hour in the dark at room temperature followed by counterstaining with DAPI (1:20.000 in PBS) for 5 min. Antibodies used include ms/rb anti-Olig2 (AB9610; 1:500; Millipore), ms anti-Ki67 (550,609; 1:75, BD Biosciences) and rb anti-Casp3 (1:300, Cell Signaling) as well as rb/gt anti-ms-Alexa488, gt anti-ms Alexa555 and gt anti-rb Alexa647 conjugated secondary antibodies (1:1000, Invitrogen, ThermoFisher Scientific). For negative controls, cells were incubated with the secondary antibody only. Pictures were taken with Zeiss Observer at a 20x magnification. Quantitative analysis was performed with the help of ImageJ by counting at least 300 Olig2-positive cells in at least 10 different visual fields.

### Generation of human induced primary neurons

iPNs were generated as previously described (30). In brief, renal proximal tubule epithelial cells (RPTECs) were isolated from the urine of participants. After cultivation, RPTECs were transfected via electroporation with the episomal plasmids pCXLE-hOCT3/4-shp53-F, pCXLE-hSK and pCXLE-hUL (Addgene). Resulting induced pluripotent stem cell (iPSC) colonies were transferred into a free-floating state, forming embryoid bodies (EBs). Differentiation into meso- and entoderm was inhibited by using 10 µM SB431542 (Biozol) and 5 µM dorsomorphin (Sigma). After inhibition, EBs were transformed into adherent state again by transferring EBs on 0.002% poly-L-ornithine (Sigma) and 10 µg/ml laminin (Sigma) coated dishes. For the induction of the formation of neural rosettes, EBs were cultivated in neural stem cell (NSC) medium (DMEM/F12 GlutaMAX™ (Thermi Fisher Scientific), supplemented with 20 µg/l insulin (Sigma), 1.6 g/l L-glucose (Applichem), 1 µl/ml B27™ supplement (Life Technologies), 1 µl/ml N2 supplement (Life Technologies), 10 ng/ml basic fibroblast growth factor (PAN Biotech), 10 ng/ml epidermal growth factor (PAN Biotech). After the emergence of neuronal rosettes, neuronal progenitor cells (NPCs) were isolated from donut-shaped structures and transferred to another dish. For the differentiation of NPCs to iPNs, medium was changed to neuronal differentiation medium composed of DMEM/F12 GlutaMAX™, supplemented with 50 µg/ml L- ascorbic acid (Carl Roth), 50 µg/ml apo-transferrin (Sigma), 2x B27™-, and 2x N2-supplement, and cells were initially treated with 500 ng/ml sonic hedgehog (SHH; Peprotech) and 2 µM retinoic acid (RA; Sigma) for the induction of differentiation. Afterwards, neuronal medium was supplemented with 10 ng/ml brain-derived neurotrophic factor (BDNF; Peprotech) and 20 ng/ml glial-derived neurotrophic factor (GDNF; Peprotech) for maintenance and maturation. iPNs were used for experiments at the age of 21-23d.

### Cell death analysis of iPNs

8,000-10,000 cells/well were plated onto a 96-well plate, 14-16 days after induction of neuronal differentiation. Cells were treated with 1.5 mM galactose for 24 h, 48 h, or 72 h or with 50 µM AGE-BSA or control-BSA for 48 h. For cell death analysis, cells were treated, except for the control condition, with 5 µM H_2_O_2_ for 2 h. For this time neuronal medium was replaced with DPBD+/+.

### Staining of iPNs

Staining was performed after treatment incubation by changing the medium to DMEM F12/Glutamax (Gibco) + 4 µg/ml Höchst (Thermo Fisher) for 1.5 h to stain the nuclei. A subsequent staining of dead cells by 5 µl/ml 7AAD (eBiosciece) in DMEM F12/Glutamax was executed for 10 min at 37°C. After incubation, the well plate was analyzed under an inverse fluorescence microscope. Images were acquired at 20,000 x magnification. Randomly, three visual fields were obtained per well. Per condition a minimum of three wells was analyzed. Total cell amount per visual field (Höchst+) and dead cells (Höchst+/7AAD+) were manually counted using the cell counter tool from ImageJ.

### Microglia isolation and analysis

Microglia were isolated from brain tissue of EAE diseased mice (d20 p.i.) via magnetic cell separation (MACS, Miltenyi Biotech) using CD11b microbeads. Microglia were identified as CD11b+CD45- cells via flow cytometry and analyzed for CD69 and MHCII expression. Cells were cultured in MACS Neuro-Medium (Miltenyi Biotech) supplemented with 2% MACS NeuroBrew-21 (Miltenyi Biotech) at a density of 2x10^5 cells/well in poly-L-Lysine (0.01%, Sigma) coated 12 well plates for 24 h. Supernatants were analyzed for the secretion of IL-1β, IL-6 and TNFα (ELISA DuoSet, R&D).

### mRNA expression analysis

Spinal cord tissue was homogenized in RLT buffer with an Ultra-Turrax for 30s. Total RNA was isolated using the RNase Mini Kit #A2791 (Qiagen) following the manufacturer’s instructions. Reverse transcription was performed by using the GoScript Reverse Transcription Mix Oligo(dT) (Promega). PCR reactions were performed at a 5 μl scale on a qTower 2.0 real time PCR System (Analytic Jena, Germany) in triplicates. Relative quantification was performed by the ΔΔCT method, normalizing target gene expression on β-Actin as housekeeping gene. The following primer (TaqMan^®^, Thermo Fisher Scientific) were used: *Ager* Mm01134790_g1, *Nos2* Mm00440502_m1, *Nrf2* (Nfe2l2) Mm00477784_m1, *Actb* Mm00607939_s1.

### Statistical analysis

Statistical analysis was performed using GraphPad Prism (GraphPad Software Inc., La Jolla, CA). All *in vitro* and *ex vivo* data were analyzed by one-way ANOVA followed by Tukey’s post-test, unpaired t-test or Wilcoxon rank sum test after checking for normal distribution (unless indicated otherwise in the legends). EAE data were analyzed by Mann–Whitney U test. Data are presented as mean ± SEM; *p< 0.05, **p< 0.01, or ***p<0.001 were considered to be statistically significant.

## Results

### A diet rich in galactose exacerbates the clinical symptoms in MOG-EAE

**Figure 1:**
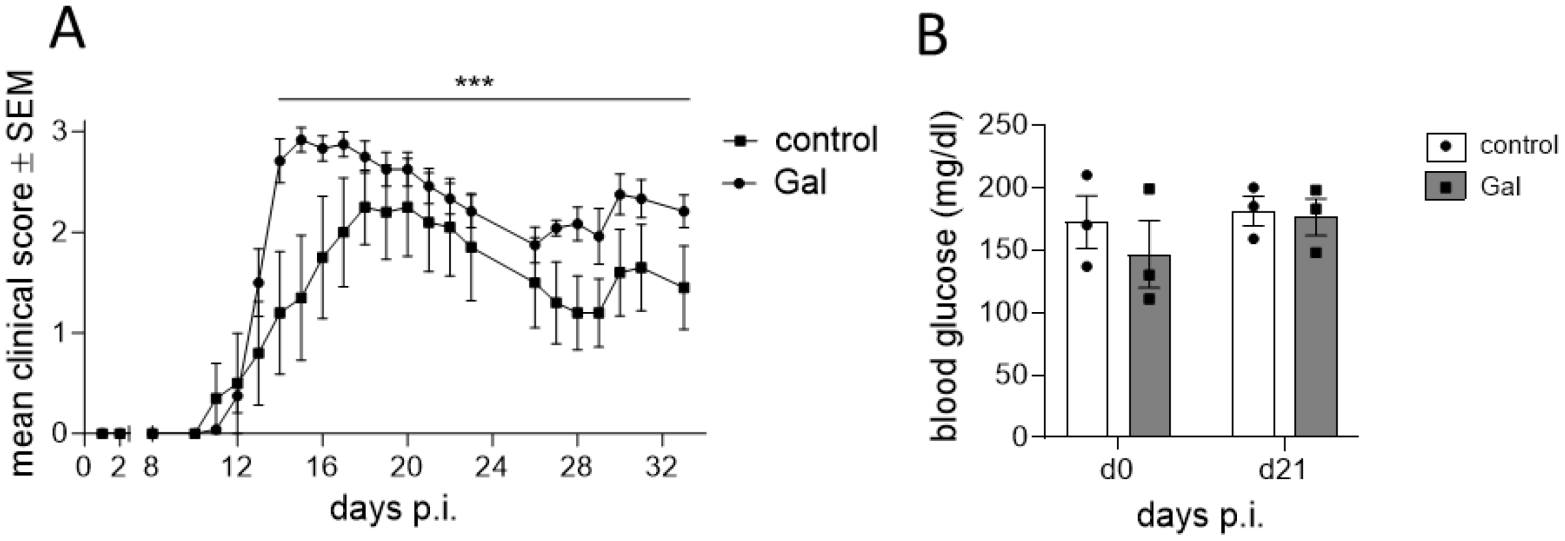
A diet rich in galactose exacerbates the clinical symptoms in MOG-EAE. C57BL/6N mice with an age of 11.9 weeks ± 4 days were adapted to a galactose-rich diet 14 days prior to active immunization and during MOG-EAE. **(A)** Clinical course of MOG-EAE (controls: n=5, galactose-diet: n=6). Mice were daily scored for clinical signs on a 5-point scale. **(B)** Blood glucose levels were measured after 14 days of galactose feeding prior to active immunization (d0) and at the maximum of MOG-EAE (d21; n=3 mice per group and time point). All data are represented as mean ± SEM; **(A)** ***p<0.001 using a Mann-Whitney test, **(B)** data were analyzed by two-way ANOVA.

To examine the impact of a galactose-rich diet on the course of EAE, young C57BL/6N mice were administered a diet consisting of a 10% galactose in drinking water. This diet was started 14 days prior to active induction of MOG_35-55_-EAE and disease severity was compared to control mice receiving normal drinking water. The galactose-rich diet significantly worsened disease symptoms in the effector phase and impaired the recovery in the chronic phase of EAE compared to controls (**Figure 1A**). No major differences between both groups were observed in terms of disease incidence (**Table 1**) or mortality (data not shown). As changes in the diet may affect physical and metabolic parameters, mice were also monitored for changes in body weight and blood sugar levels. A galactose-rich diet was neither associated with significant body weight changes during the 14-day adaptation period prior to EAE induction nor in the period after immunization compared to the control (**Table 1**). Blood glucose levels were similar in both groups prior to EAE induction and at the maximum of the disease (**Figure 1B**).

**Table 1:** A diet rich in galactose does not affect disease incidence or weight change during MOG-EAE.

		control	Gal
EAE incidence	[sick/total]	5/6	6/7
body weight d-14	[g] mean ± SEM	24.8 ± 1.2	26.9 ± 0.8
body weight d0	[g] mean ± SEM	25.9 ± 0.9	27.5 ± 1.3
body weight d33	[g] mean ± SEM	25.5 ± 1.1	27.8 ± 1.9

Body weights of mice receiving either a control or a galactose-rich diet were monitored starting 14 days prior to active immunization and during the course of EAE. Mean weight ± SEM is given for d-14, the day of immunization (d0) and d33 post immunization.

### A galactose-rich diet significantly increased demyelination and impaired the neuro-axonal integrity during MOG-EAE

**Figure 2:**
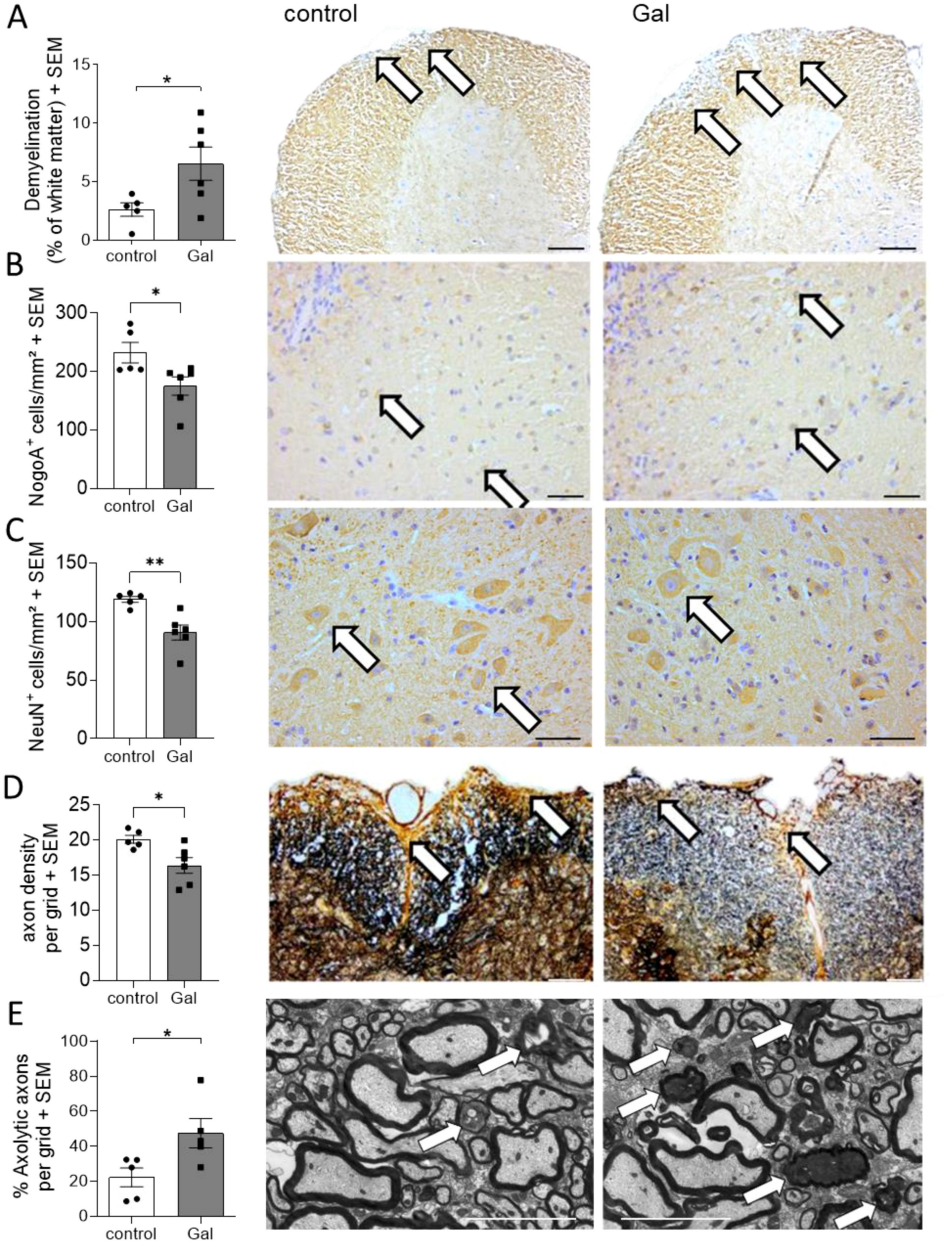
A galactose-rich diet significantly increased demyelination and impaired the neuro-axonal integrity during MOG-EAE. C57BL/6N mice were adapted to a galactose-rich diet 14 days prior to active immunization and during MOG-EAE. Histological analyses were performed on spinal cord cross sections from control or galactose-treated (Gal) mice suffering from chronic EAE (34 days p.i., control n=5, Gal n=6) for **(A)** demyelination with CNPase staining, IHC-staining of **(B)** oligodendrocytes, **(C)** anterior horn neurons, and **(D)** axonal densities determined by Bielschowsky silver staining. **(E)** Analysis of axolytic axons using electron microscopy (n=5 per group). Left: Scale bars depict mean ± SEM. Right: Representative images of the respective staining. Scale bars are 400µm in **(A, B)**, 200µm **(C, D)** and 5µm in **(E)**. White arrows show the demyelinated regions in **(A)**, positive cells in **(B, C)**, axons in **(D)** or axolytic axons in **(E)**. *p<0.05, **p<0.01 using an unpaired t-test.

To analyze potential mechanisms of galactose-action in MOG-EAE, we performed histopathological analyses on spinal cord cross sections from control treated mice and mice receiving a galactose-rich diet during the chronic phase of the disease on day 34 post immunization (p.i.). In accordance with the observed exacerbation of clinical symptoms, CNPase staining revealed a more pronounced demyelination in mice receiving a galactose-rich diet (**Figure 2A**). Moreover, NogoA+ mature oligodendrocytes were significantly reduced by high galactose intake (**Figure 2B**). Further histological analysis showed a significant loss of NeuN^+^ neurons in the anterior horn of these mice (**Figure 2C**). We next studied the effect of a galactose-rich diet on the neuro-axonal integrity during EAE. Bielschowsky’s silver impregnation of spinal cord cross sections revealed a reduced axonal density in mice receiving a galactose-rich diet compared to the control group (**Figure 2D**). Using electron microscopy, we further analyzed the effect of galactose on axonal integrity and detected an increased number of axolytic axons (**Figure 2E**). In order to exclude an effect of a galactose-rich diet on CNS cells in the absence of autoimmune processes, we also histologically analyzed spinal cord tissue of naïve young mice (11.2 weeks ± 5 days) receiving a galactose-rich diet for 7 weeks. No obvious differences in the number of oligodendrocytes, neurons or astrocytes were observed as compared to naïve mice fed a normal diet (**Supplementary Figure 1**).

### A galactose-rich diet does not affect immunological processes during MOG-EAE

**Figure 3:**
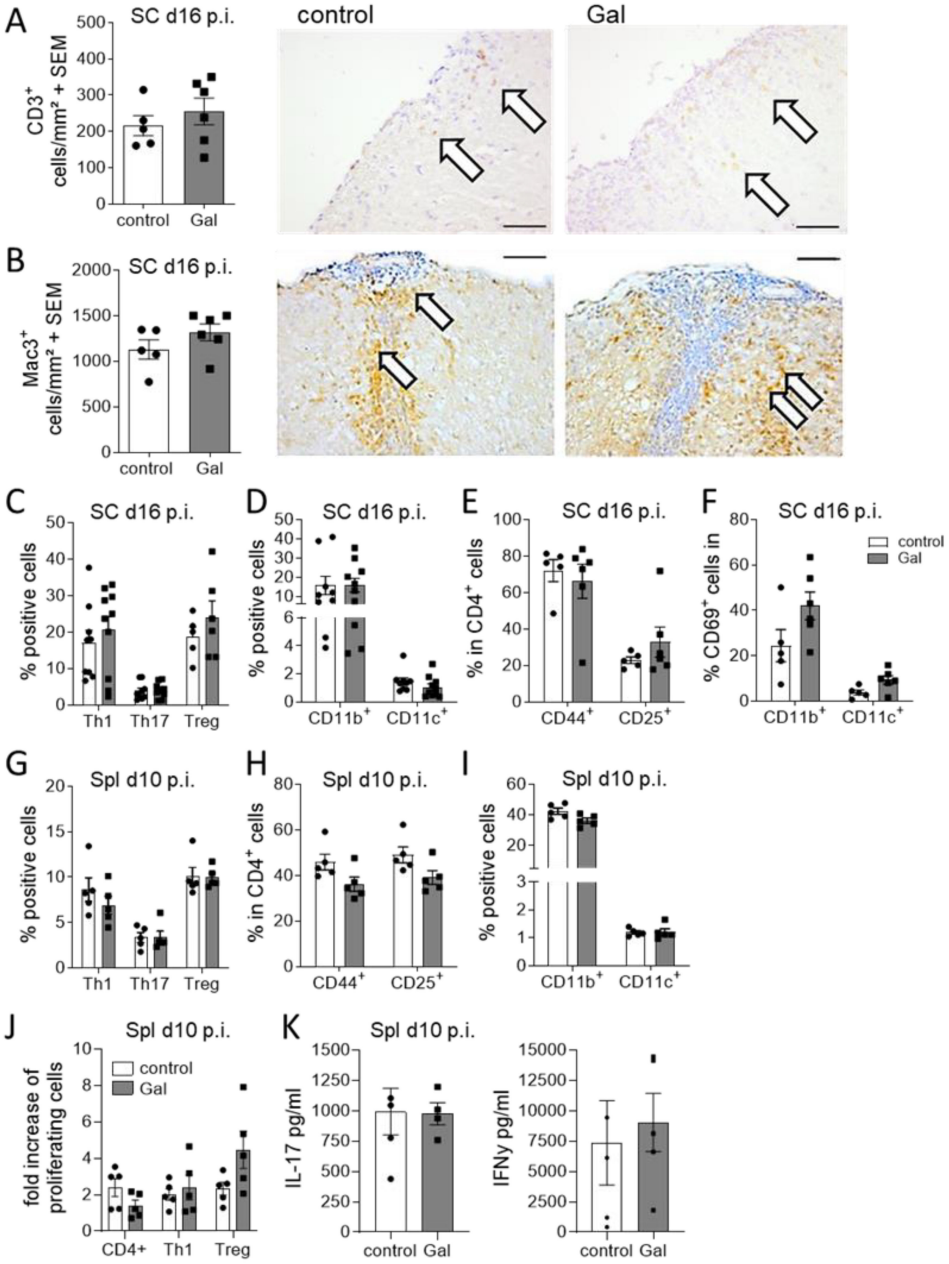
A galactose-rich diet does not affect immunological processes during MOG-EAE. C57BL/6N mice were adapted to a galactose-rich diet 14 days prior to active immunization and during MOG-EAE. **(A, B)** Histological analysis of spinal cord cross sections for **(A)** CD3+ T cells and **(B)** Mac3+ macrophages/microglia at the maximum of EAE (16 days p.i., control n=5, Gal n=6). Right: Representative images. Scale bars are 200µm. White arrows mark positive cells. **(C, D)** Flow cytometry analysis of spinal cord infiltrating cells isolated on day 16 of EAE for IFNγ+ Th1 cells, IL-17A+ Th17 cells and FoxP3+ Treg cells and **(D)** CD11b+/CD11c+ APCs (Th1, Th17, CD11b, CD11c control n=9, Gal n=10; Treg control n=5, Gal n=6). **(E)** Activation of spinal cord infiltrating CD4+ T cells was analyzed by flow cytometry analysis of CD44 and CD25 expression in CD4+ T cells (n=5 per group). **(F)** Activation of spinal cord CD11b+/CD11c+ cells was analyzed by flow cytometry analysis of CD69 expression in CD11b+/CD11c+ cells in the spinal cord in day 16 p.i. (n=5 per group). **(G, H)** Flow cytometry analysis of splenic cells isolated on day 10 of EAE for **(G)** IFNγ+ Th1 cells, IL-17A+ Th17 cells and CD25+FoxP3+ Treg cells and **(H)** CD11b+/CD11c+ APCs. (n=5 per group). **(I)** Activation of splenic CD4+ T cells was analyzed by flow cytometry analysis of CD44 and CD25 expression in CD4+ T cells (n=5 per group). **(J, K)** Splenocytes were isolated on day 10 of EAE from control and galactose treated mice and *in vitro* restimulated with MOG peptide for 48h and 72h. **(J)** Proliferation of CD4+ T cells, IFNγ secreting Th1 cells or FoxP3 expressing Treg cells was analyzed via flow cytometry as fold increase of proliferating cells upon antigen re-stimulation compared to control treated cells after 72h. (n=5 per group). **(K)** Cytokines in cell culture supernatants were measured by ELISA after 48h. All data are presented as mean ± SEM. Scale bars depict mean ± SEM. Data were analyzed by unpaired t-test or two-way ANOVA.

In contrast to the evident effects of galactose on the oligodendroglial and neuronal compartment, no changes in numbers of infiltrating CD3+ T cells and Mac-3+ macrophage/microglia were observed at the maximum of EAE (**Figures 3A, B**). Further flow cytometry analysis of spinal cord infiltrating cells at day 16 of EAE revealed no obvious differences between mice on a galactose-rich diet and controls. There were neither effects on the frequencies of Th1, Th17 and Treg cells (**Figure 3C**) nor on CD11b^+^/CD11c^+^ antigen-presenting cells (**Figure 3D**). Additional analysis of T cell activation also revealed no differences in CD44+ and CD25+ expression (**Figure 3E**). CD69 expression in CNS infiltrating macrophages and brain resident microglia also revealed no differences in galactose fed mice compared to the controls (**Figure 3F**). To additionally investigate potential effects of galactose on the immune system, we also performed flow cytometry analysis of splenic cells prior to the onset of EAE symptoms on day 10 p.i. as well as during acute stages of the disease on day 16 p.i. A galactose-rich diet did not affect the frequency of splenic Th1, Th17 or Treg cells (**Figure 3G**), T cell activation (**Figure 3H**) or CD11b^+^/CD11c^+^ antigen-presenting cells (**Figure 3I**) prior to disease onset or at later stages (data not shown). Moreover, upon antigen-specific restimulation of splenocytes *in vitro*, neither the proliferation of lymphocytes (**Figure 3J**) nor the secretion of the pro-inflammatory cytokines IFNγ and IL-17 showed any difference between cells from mice receiving a control or galactose-rich diet (**Figure 3K**).

### Galactose enhances the formation of AGEs that affect neuronal and oligodendroglial cell viability *in vitro*

**Figure 4:**
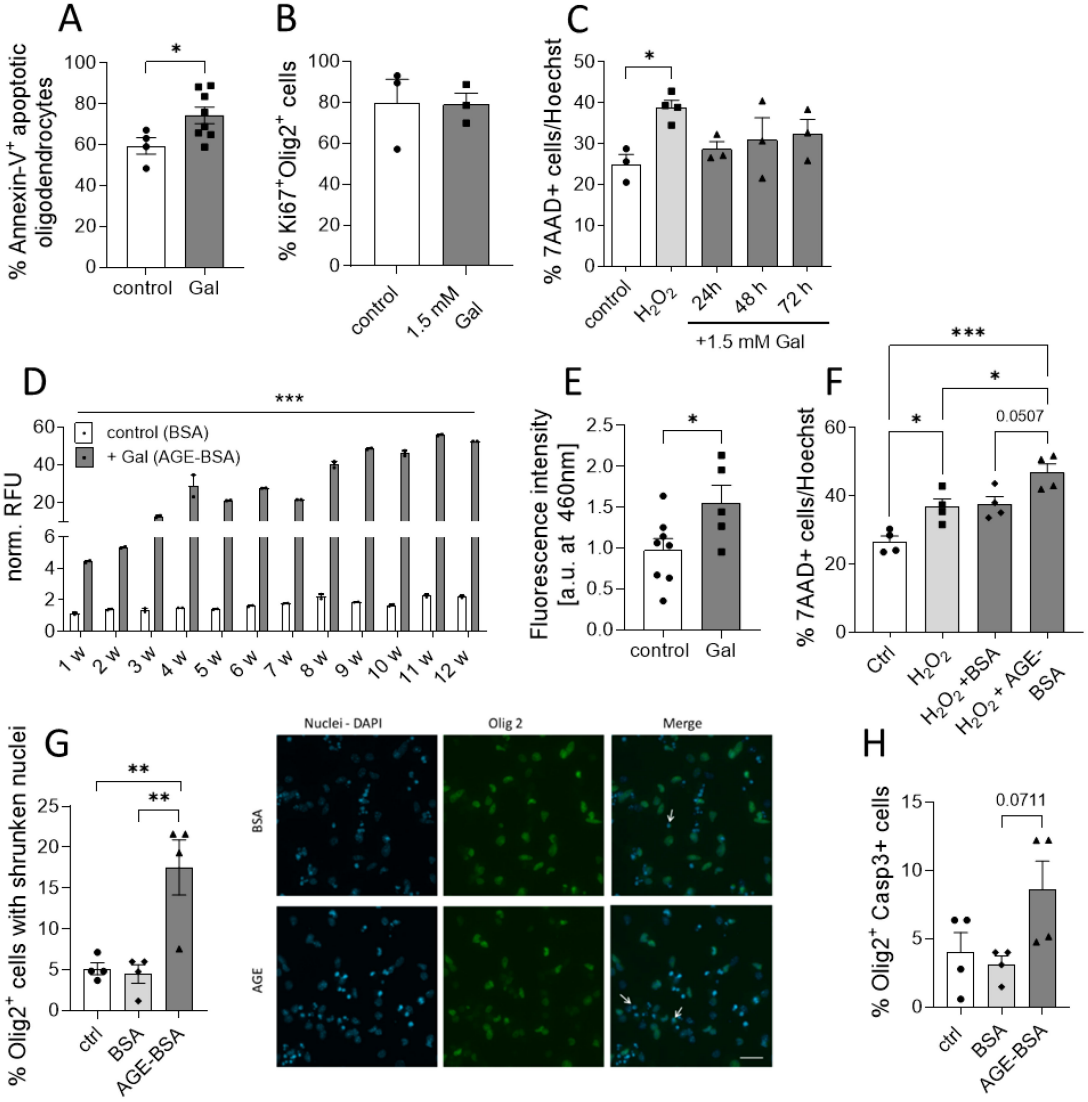
Galactose enhances the formation of AGEs that affects neuronal and oligodendroglial cell viability. **(A)** C57BL/6N mice were adapted to a galactose-rich diet 14 days prior to active immunization and during MOG-EAE. Oligodendrocytes (O4+) were isolated via magnetic cell separation on day 20 of EAE and stained with Annexin-V and viability dye for the analysis of cell apoptosis. The frequency of Annexin-V+ cells in O4+ cells was analyzed by flow cytometry (control n=5, Gal n=8). **(B)** Percentage of Ki67+ Olig2+ proliferative cells in oligodendroglial cell cultures after incubation with or without 1.5 mM galactose for 3 days determined by immunofluorescence staining (n=3). **(C)** Percentage of apoptotic cells in human neuronal iPSCs cultures treated with 1.5mM galactose for 24h, 48h and 72h (n=3-4 from two independent experiments). **(D)** Incubation of BSA (10 mg/ml) without galactose (control) or with 0.5 M D-galactose (AGE-BSA) and weekly determination of the formation of AGE-BSA over a 12 week (w) period after dialysis by measurement of autofluorescence (n=3, one representative experiment is shown). **(E)** Determination of fluorescent AGEs in protein isolated from spinal cord on day 16 of MOG-EAE of control diet mice compared to mice on a galactose-rich diet (control n=8; Gal n=5). **(F)** Percentage of apoptotic cells in human neuronal iPSCs cultures treated with H_2_O_2_, H_2_O_2_+BSA or H_2_O_2_+50 µg/ml AGE-BSA for 48 h (n=4 from two independent experiments). **(G)** Percentage of Olig2+ cells with apoptotic nuclei in oligodendrocyte cultures after treatment with 50 µg/ml AGE-BSA or BSA alone for 72 h assessed by immunofluorescence staining (n=4) and representative images (arrows indicate Olig2+ cells with shrunken nuclei). **(H)** Percentage of Caspase-3+ cells in oligodendroglial cell cultures after treatment with 50 µg/ml AGE-BSA or BSA alone for 72 h assessed by immunofluorescence staining (n=4). Scale bars depict mean ± SEM; *p<0.05, **p< 0.01, ***p<0.001 using an unpaired t test in **(A, B, E)** or an ordinary one-way ANOVA in **(C, D, F–H)**.

Our histopathological observations suggested that galactose might impair the neuro-axonal integrity during MOG-EAE by decreasing oligodendrocyte and neuron numbers. Additional analysis of *ex vivo* obtained oligodendrocytes from EAE diseased mice revealed an enhanced Annexin-V uptake in cells isolated from galactose-fed mice compared to the controls, suggesting a potential effect of increased galactose concentrations on cell apoptosis (**Figure 4A**). We therefore investigated a potential direct effect of elevated galactose concentrations in *in vitro* culture assays. The addition of 1.5 mM galactose, mimicking a high yet still physiological blood concentration, the proliferation of isolated O4^+^ oligodendrocytes as assessed by Ki67 staining (**Figure 4B**). Moreover, human iPNs derived from iPSCs were cultured in the presence of 1.5 mM galactose for one to three days. Yet, the addition of galactose did not impact on cell survival in iPNs as compared to H_2_O_2_ treatment as a positive control (**Figure 4C**). Given our findings on the missing effect of galactose on CNS cells *in vitro*, we hypothesized that not galactose itself, but some reactive products may be responsible for the exacerbation of clinical symptoms and the neuroglial damage in the EAE model. Galactose is a reducing sugar and during protein glycation, amino groups of proteins may react with these sugars in a non-enzymatic reaction (31). Indeed, incubation of 10 mg/ml BSA with 0.5 M galactose at 37°C for up to 12 weeks resulted in the formation of advanced glycation end product (AGE)-coupled BSA as measured by auto-fluorescence of pentosidine as a common AGE variant (**Figure 4D**). While relative fluorescent units of control-BSA were at about one to two after 12 weeks of incubation, relative fluorescent units of BSA incubated with galactose were 20-fold higher after 5 weeks of incubation and reached almost 50 units after 12 weeks of incubation, demonstrating an increased AGE production due to galactose addition (**Figure 4D**). Interestingly, fluorescence measurements of spinal cord protein isolated from EAE diseased mice on the maximum of EAE also revealed higher fluorescent units in mice receiving a galactose-rich diet compared to the controls (**Figure 4E**). These data imply an increased concentration of fluorescent AGEs in the spinal cord of galactose-treated mice, indicating a potential role of AGEs in the observed neuropathological effects during MOG-EAE. We therefore tested the impact of AGE treatment on cell survival in human iPSC-derived iPNs and murine oligodendroglial cell cultures. iPSC-derived iPNs were treated with H_2_O_2_ to induce cell death. While the combined addition of H_2_O_2_ and control-BSA did not further increase neuronal cell death compared to H_2_O_2_ treatment alone, the addition of H_2_O_2_ together with galactose-induced AGEs significantly aggravated cell survival compared to H_2_O_2_ treated neurons (**Figure 4F**). Yet, the difference on neuronal cell death between H_2_O_2_+BSA and H_2_O_2_+AGE-BSA treated cells is not statistically significant when comparing all four groups (p=0.0507). However, the frequency of dead cells in H_2_O_2_+BSA treated cultures is comparable to the frequency of dead cells in H_2_O_2_ cultures (H_2_O_2_: 36.8 ± 3.7% vs. H_2_O_2_+BSA: 37.5 ± 3.8%). In contrast, the treatment with H_2_O_2_+AGE-BSA increased the cell death to 48.5 ± 3.4%. Hence, H_2_O_2_+AGE-BSA treatment increases neuronal cell death by around 32% compared to H_2_O_2_ or 29% compared to the H_2_O_2_+BSA treatment. Similar to the deleterious effect of galactose-induced AGEs on neuronal cell viability *in vitro*, oligodendrocytes cultured in the presence of galactose-induced AGEs showed enhanced apoptosis compared to control-BSA treated cells. More precisely, treatment of O4^+^ oligodendrocytes with galactose-induced AGEs for 72 h resulted in a more pronounced cell apoptosis compared to BSA treatment as assessed by counting Olig2^+^ cells with shrunken cell nuclei (**Figure 4G**) or caspase-3 positive Olig2+ cells (**Figure 4H**). Twice as many cells presented features of apoptosis in comparison to control-BSA treated cells, strengthening our concept that galactose-induced AGEs adversely affect neuronal and oligodendroglial cell viability *in vitro* and during MOG-EAE. We performed additional experiments to identify a potential mechanism of enhanced oligodendrocyte apoptosis due to increased galactose intake. Yet, we observed no differences in the expression of the receptor for AGEs (RAGE) in the CNS or in oligodendroglial cells during MOG-EAE (**Supplementary Figures 2A, B**). The analysis of nitrite levels, nitric oxide synthase 2 (Nos2) and Nuclear factor erythroid 2-related factor 2 (Nrf2) as markers for oxidative stress revealed no differences in galactose-fed mice compared to the controls (**Supplementary Figures 2C-E**). Moreover, the analysis of microglia cells isolated from EAE-diseased mice receiving either control or a galactose-rich diet demonstrated no differences in both groups, arguing for a direct effect of galactose or galactose induced AGEs on oligodendroglial cells during neuroinflammation.

## Discussion

In this study, we show that a galactose-rich diet exacerbates EAE severity in its acute phase and leads to impaired recovery in the chronic phase of neuroinflammation. Interestingly, flow cytometry analysis revealed that this was not accompanied by enhanced immunological responses in the spleen, neither in the pre-clinical phase of EAE nor at the disease maximum. In line with this observation, galactose induced EAE severity was not mediated by increased immune cell infiltration in the spinal cord at the maximum of the disease as assessed histologically and via flow cytometry, suggesting that the observed differences in the course of disease are not primarily driven by quantitative changes in immunological processes but rather due to early axonal loss (32, 33). Of note, histological analyses revealed that a galactose-rich diet significantly increased demyelination and impaired neuro-axonal integrity as evident from reductions in proportions of myelinated area, numbers of neurons and axonal densities as well as an increased proportion of axolytic axons and oligodendrocyte apoptosis. However, galactose treatment of iPSC-derived human iPNs did not affect cell survival *in vitro*. Additionally, galactose treatment of oligodendroglial cells did not cause an altered proliferation capacity *in vitro*, indicating that the observed *in vivo* effects of galactose on myelin and neuro-axonal integrity may not be primarily driven by the sugar itself. We suggested that galactose may lead to the formation of advanced glycation end products (AGEs). These heterogeneous macromolecules are formed by glycation *in vitro* and *in vivo* in the so called non-enzymatic Maillard reaction between reactive sugars such as galactose and amino groups, lipids or nucleic acids (34, 35).

AGEs also play a key role in galactose-mediated aging models: Rodents treated chronically with galactose have been described to develop a phenotype resembling features of the natural aging process, including cognitive dysfunction, neurodegeneration and shortened life span (36). Therefore, this model is commonly used for age research and drug testing (36, 37). However, for aging models, galactose is given subcutaneously or intraperitoneally for up to 16 weeks. In contrast, we here assessed the impact of a galactose enriched diet applied orally via the drinking water for only 5 to 7 weeks. The mechanism leading to the features of aging is not completely understood, but there is accumulating evidence for an important role of oxidative stress and AGEs (35, 37). AGEs are known to physiologically accumulate throughout the body with increasing age and have been proposed to contribute to age-related stiffening of heart, tendons, skeletal muscle and blood vessels (35). They are also associated with a number of neurodegenerative diseases, as there is evidence on increased beta-amyloid formation by AGEs in Alzheimer’s disease (38), increased aggregation of alpha-synuclein, and increased AGE-levels in the frontal cortex of Parkinson’s disease patients (39). Some clinical studies also implicate AGEs in EAE and MS pathogenesis and suggest their potential use as biomarker. One study reported an increase of AGE levels in the plasma and brain of MS patients (40, 41). In addition, Wetzels and co-workers showed increased AGE levels in MS lesions and AGE production by astrocytes which may affect microglia (42).By incubation of BSA with galactose, we were able to generate AGEs *in vitro* as assessed by fluorescence measurement, which is well in line with previous findings (43). Furthermore, we showed a time-dependent increase in AGE concentration during the incubation period. To check whether a galactose rich diet leads to increased AGE-levels during neuroinflammation *in vivo*, protein was isolated from spinal cords of EAE diseased mice. Here, we found higher AGE-associated fluorescence in galactose-treated animals compared to controls. Next, the effect of galactose-derived AGEs was tested on iPSC-derived neurons and in oligodendroglial cell cultures. In line with previous studies, reporting a toxic effect of AGEs on rat cortical neurons (29, 44), we found an increased rate of cell death in AGE-treated neuronal cells as well as oligodendroglial cultures. Regarding the role of AGEs in oligodendrocyte culture, to our knowledge, no data have been published so far. However, there are studies supporting the relevance of receptors of AGEs (RAGE) to oligodendrocyte function and neuronal regeneration in spinal cord injury (45), which describe surface expression of RAGEs on oligodendrocytes as well as its shedding upon oxidative stress (46).

Increased neuronal and oligodendroglial cell death by AGE treatment and galactose diet, respectively, may be mediated by RAGE signaling. RAGE is expressed on monocytes/macrophages, neurons, neutrophils, lymphocytes, vascular endothelial cells and oligodendrocytes (46, 47). Its expression can be induced by accumulation of ligands such as AGEs, amyloid β peptide, DNA-binding protein high mobility group box-1 (HMBG1) and S100/calgranulins as well as in the presence of inflammatory mediators (48). The toxic effects of AGEs on neurons were mainly attributed to increased oxidative stress after binding to RAGE (29, 49). Downstream signaling of RAGE leads to NF-kB activation, proposing a role of AGE-RAGE interaction in inflammation and immunological processes (50). The relevance of RAGE in the context of MS and EAE was supported by an upregulation of RAGE in active MS lesions as well as in spinal cord tissue in the EAE model. Blockade of RAGE with soluble RAGE (sRAGE), the cleaved variant of RAGE, was able to prevent activation of membrane-bound RAGE and partially protected animals from EAE (51). Accordingly, sRAGE levels were reduced in the serum and CSF of MS patients (52, 53).

The observed increased damage in oligodendroglial and neuronal cells upon AGE treatment as well as dietary galactose, may be attributed to a RAGE-mediated increase in oxidative stress and NF-κB activation followed by secretion of pro-inflammatory cytokines. Yet, we detected no differences in RAGE expression in the spinal cord of EAE diseased mice receiving either a control or galactose-rich diet. Moreover, oligodendrocytes isolated from EAE-diseased mice demonstrated a similar RAGE expression in both groups, suggesting no significant involvement of RAGE expression during galactose-induced oligodendroglial apoptosis (**Supplementary Figures 2A, B**). The analysis of nitric oxide synthase 2 (Nos2/iNOS) as a key enzyme involved in the production of nitrogen species, as well as Nuclear factor erythroid-2-related factor 2 (Nrf2) as a key regulator of anti-oxidant pathways revealed no differences in galactose fed mice compared to the controls during MOG-EAE. Coinciding with this, we detected comparable nitrite levels in the serum of EAE diseased mice either receiving galactose in control, suggesting no relevant involvement of oxidative stress in our experiments (**Supplementary Figures 2C-E**). However, a RAGE-specific blockade *in vitro* or anti-RAGE treatment during MOG-EAE may yield even more conclusive results. Another detrimental mechanism may include activation of microglia by AGEs (54) leading microglia-driven oligodendrocyte damage (55). Yet, we detected no differences in the activation status of microglia isolated from EAE diseased mice receiving either a control or galactose-rich diet. We observed comparable levels of CD69 and MHCII expression as well as inflammatory cytokine secretion, suggesting no involvement of microglia activity here (**Supplementary Figures 2F-K**). Yet, additional experiments will be necessary to identify a conclusive mechanism of enhanced oligodendrocyte apoptosis due to increased galactose intake.

In summary, combining our findings with those from other groups, we assume that not galactose itself, but rather AGEs are responsible for enhanced EAE severity *in vivo* after a galactose-rich diet. We were able to correlate increased disease severity with reduced axonal and neuronal density in the spinal cord, as well as increased demyelination, while immunologic parameters in spleen and spinal cord remained unaltered. Although the precise mechanisms of galactose-driven EAE severity remain unclear, we suggest a detrimental effect of galactose-induced AGEs on oligodendrocytes and neuronal cells, leading to loss of myelin integrity and axons *in vivo* and increased cell apoptosis *in vitro* and *ex vivo*. The present study links neurodegeneration observed in galactose-induced aging with enhanced neuronal and oligodendroglial damage following galactose rich diet in neuroinflammation. Moreover, it provides support for a role of AGE in susceptibility for neuronal damage. Hence, it appears promising to conduct further investigations into the effects of AGEs on MS and other neurodegenerative diseases, especially in light of high consumption of dairy products, and escalating consumption of processed foods with substantial quantities of AGEs (35). Ultimately, this may open up new avenues for establishing dietary guidelines or dietary supplements for patients serving as additional therapeutic measures in neurological diseases.

## Data Availability

The original contributions presented in the study are included in the article/**Supplementary Material.** Further inquiries can be directed to the corresponding author.
