# Supplementary material for: Dietary galactose exacerbates autoimmune neuroinflammation via advanced glycation end product-mediated neurodegeneration
Source: Front Immunol. 2024 Aug 9;15:1367819. doi: 10.3389/fimmu.2024.1367819 (PMC11341352; doi:10.3389/fimmu.2024.1367819)
Supplement: Supplementary file 1 [file DataSheet_1.pdf]

Supplementary Figures

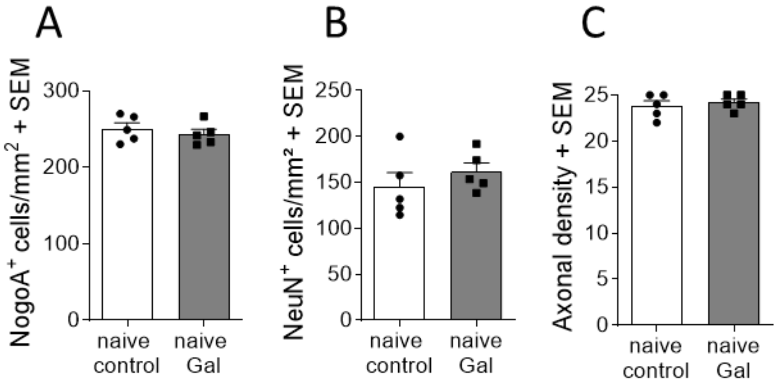

Supplementary Figure 1. A galactose-rich diet does not affect the neuro-axonal integrity in naïve mice. C57BL/6N mice with an age of 11.2 weeks ± 5 days were adapted to a galactose-rich diet for 7 weeks. Histological analyses were performed on spinal cord cross sections from control (naive control) or galactose-treated (naive Gal) mice for (A) NogoA+ oligodendrocytes, (B) anterior horn neurons, and (C) axonal densities determined by Bielschowsky silver staining. (n=5 per group). Scale bars depict mean ± SEM. Data were analyzed by an unpaired t-test.

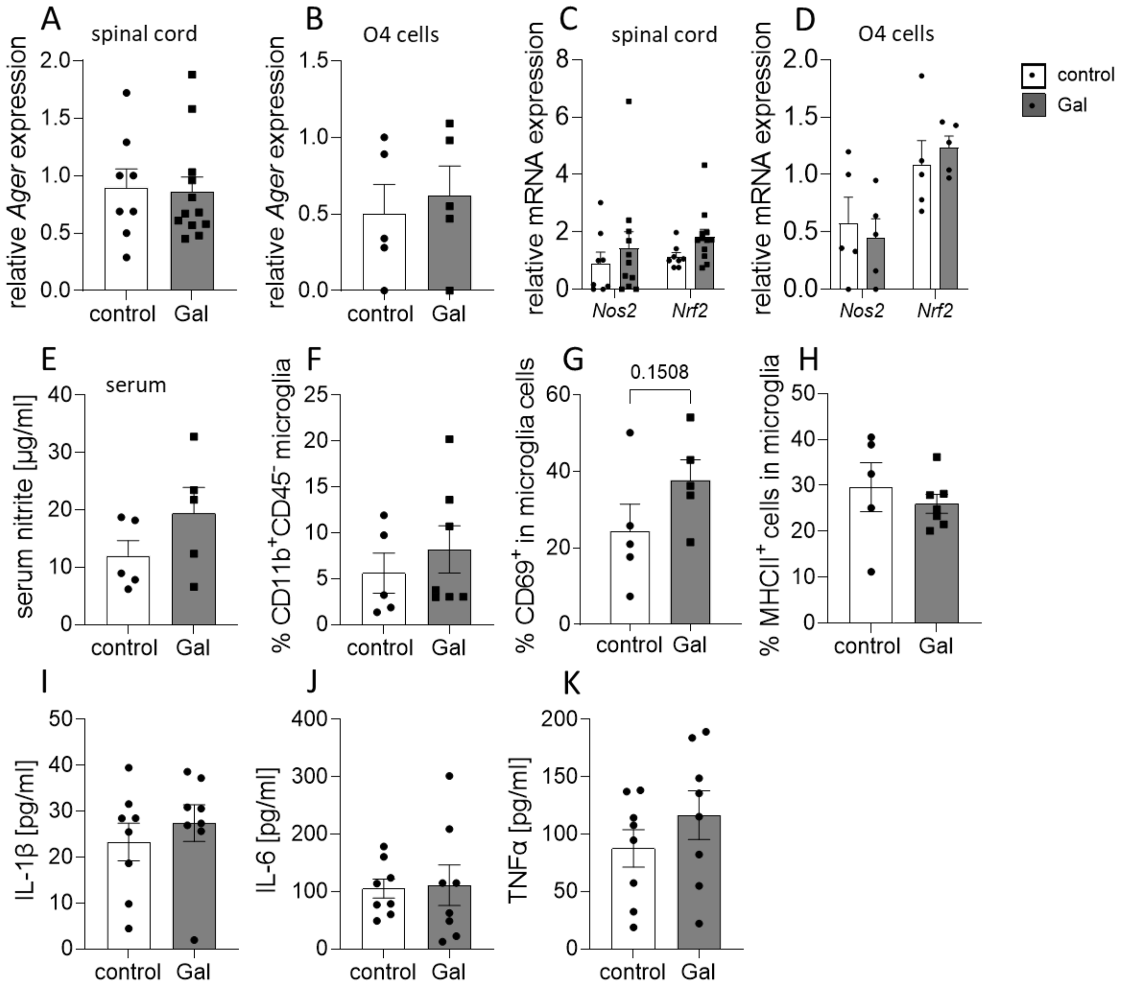

Supplementary Figure 2. Galactose intake does not affect RAGE expression, oxidative stress responses or microglia activity during MOG-EAE. C57BL/6N mice were adapted to a galactose-rich diet 14 days prior to active immunization and during MOG-EAE. (A, B) RAGE (*Ager*) mRNA expression was analyzed in (A) spinal cord tissue on day 16 of EAE (control n=8, Gal n=12) and (B) oligodendrocytes isolated from EAE diseased mice (day 20 p.i.) receiving either control or a galactose-rich diet (n=5 per group). (C, D) mRNA expression analysis of *Nos2* and *Nrf2* in (C) spinal cord tissue on day 16 of EAE (control n=8, Gal n=12) and (D) in oligodendrocytes isolated on day 20 of EAE (n=5 per group). (E) Nitrite levels were analyzed in serum samples from EAE diseased mice (d16 p.i.) receiving either a galactose-rich diet or control with a Griess-Assay (n=5 per group). (F-K) Microglia were isolated via magnetic cell separation (MACS) on day 20 of EAE and analyzed via flow cytometry for (F) viable microglia (CD11b+CD45-), (G) CD69 expression and (H) MHCII expression (control n=5, Gal n=7). (I-K) Isolated microglia were cultured in 48-well culture plates for 24h. Supernatants were analyzed for (I) IL-1β, (J) IL-6 and (K) TNFα via ELISA (n=8 per group). Scale bars depict mean ± SEM. Data were analyzed by an unpaired t-test.
